# BNT162b2 and ChAdOx1 SARS-CoV-2 Post-vaccination Side-Effects Among Saudi Vaccinees

**DOI:** 10.3389/fmed.2021.760047

**Published:** 2021-10-08

**Authors:** Ahmed N. Alghamdi, Mohammed I. Alotaibi, Adel S. Alqahtani, Daifullah Al Aboud, Ahmed S. Abdel-Moneim

**Affiliations:** College of Medicine, Taif University, Taif, Saudi Arabia

**Keywords:** COVID-19, SARS-CoV-2, COVID vaccines, post vaccination side effects, ChAdOx1 nCoV-19 vaccine, BNT162b2 mRNA COVID-19 vaccine, palpitation, menstrual cycle disturbance

## Abstract

**Background:** Vaccination against SARS-CoV-2 is important for reducing hospitalization and mortalities. Both Pfizer-BioNTech (BNT162b2) and the Oxford-AstraZeneca (ChAdOx1 nCoV-19) vaccines are used in Saudi Arabia and in many parts of the world. Post-vaccinal side effects were recorded, so we aimed to screen different complaints after vaccination among vaccinees in Saudi Arabia.

**Methods:** An online questionnaire was designed to screen the local, systemic, and allergic post vaccination reactions for vaccinees who received either one or two doses of the BNT162b2 vaccine or one dose of the ChAdOx1 vaccine. The number and percentage were recorded for each response and analyzed using cross-tab and Chi square tests. The degree of the severity of post vaccination reactions were analyzed using Roc curve. The cofactors that may affect the severity of post-vaccinal reactions including previous COVID-19 infection, age, sex, body mass index, and comorbidities were investigated.

**Results:** During our study, 4,170 individuals reported their responses: 2,601 received one dose of BNT162b2, of whom 456 completed the second dose, and 1,569 received a single dose of ChAdOx1. The side effects were reported in 85.6% of BNT162b2 vaccinees and 96.05% of ChAdOx1 vaccinees who voluntarily responded to a survey about post-vaccination side effects. The side effects were more severe in BNT162b2 than ChAdOx1. ChAdOx1 vaccinees reported mild, moderate, severe and critical side effects in 30.13, 28.62, 29.73, and 1.53%, respectively. In contrast, mild side effects were recorded among the majority of BNT162b2 vaccinees (63.92%) while moderate, severe, and critical side effects were 27.67, 7.68, and 0.72%, respectively. Both local and systemic side effects were recorded more frequently in ChAdOx1 in comparison to BNT162b2 vaccinees. Palpitation was among the new systemic side effects reported in the current study in high frequency. Abnormal menstrual cycle (delaying/increase hemorrhages or pain) was also reported in 0.98% (18/1846) of Pfizer-BioNTech and 0.68% (7/1028) of ChAdOx1 vaccinees, while deep vein thrombosis was only reported in a single case vaccinated with BNT162b2 vaccine.

**Conclusion:** Both vaccines induced post-vaccinal side effects; however, ChAdOx1 induces a higher frequency of post-vaccinal systemic side effects than BNT162b2.

## Background

The SARS-CoV-2 pandemic continues to spread worldwide, following its first emergence in December 2019. The virus induces coronavirus disease (COVID-19) that might be asymptomatic or mild in the majority of patients; however, 20% of infected patients are prone to develop severe to serious diseases with fatal consequences (1). Old age, gender, asthma, hypertension, and other cardiovascular diseases are among the risk factors of COVID-19 (2–4).

Vaccination is the most successful way to prevent the spread of many life-threatening infectious diseases. Thanks to vaccines, millions of lives are saved annually against more than 20 infectious diseases. Some countries have implemented local legislations for COVID-19 obligatory vaccination for access to private and governmental public sectors, making it mandatory for travelers prior to entry into such countries. Despite the significant number of vaccinated individuals worldwide, many people around the world are reluctant to get vaccinated against COVID-19. This fact could be due to the widespread rumors regarding the panic from the side effects that have led to a lack of trust in COVID-19 vaccines.

Starting from December 2020, many SARS-CoV-2 vaccines have been released for emergency use, including spike- gene RNA–vaccines encapsulated in lipid nanoparticles, such as BNT162b2 (Pfizer–BioNTech) and mRNA-1273 (Moderna); chimpanzee adenovirus vector vaccine harboring SARS-CoV-2 S gene (ChAdOx1- AstraZeneca), human adenovirus 26 (Ad26.COV2.S-Johnson & Johnson/Janssen), and inactivated SARS-CoV-2 vaccines (Sinopharm and Sinovac). Saudi Arabia approved BNT162b2 then ChAdOx1 for use in the vaccination protocol on 10 December 2020, and 18 February 2021, respectively. Both vaccines have proven to be safe and efficacious as evidenced by the results of clinical trials (5, 6). There is a need of up to 14 billion doses of COVID-19 vaccines to cover 70% of global coverage (7). This fact results in the recommendation of the WHO to temporary halt of giving the second dose of COVID-19 vaccine, with the aim of covering more unvaccinated people worldwide (8).

Pain, redness, swelling, fever, tiredness, headache, muscle pain, chills, and nausea are among the common side effects of the vaccines (9). Most of such side effects disappear within 2 days after vaccination (9). Other rare side effects, including vesicolous-bullous skin (10), herpes simplex or varicella zoster reactivation (11–13), nephrotic syndrome and acute kidney injury (14) were also reported. Meanwhile, serious post-vaccinal reactions, including intravascular thrombosis and thrombocytopenia are associated with both ChAdOx1 and the BNT162b2 vaccines; however, the frequency was 5-fold higher in ChAdOx1 than in BNT162b2 (15). It was found that the ChAdOx1 vaccine but not BNT162b2 enhances the production of anti-platelet factor 4 antibodies (15, 16). Accordingly, some countries in Europe suspended the use of the ChAdOx1 vaccine. However, KSA and other European countries decided to continue using the vaccine according to the WHO recommendation that benefits of using ChAdOx1 vaccine outweigh its risks (17).

On the other hand, anaphylaxis reaction has been reported among BNT162b2 vaccinees in an approximate rate of 1:200,000. Such reaction was most probably induced by the polyethylene glycol (PEG 2000 Da) (18). Interestingly ChAdOx1 vaccine contains polysorbate 80, a derivative of the PEG, which is used in a similar concentration in many vaccines including DTaP, HB vaccine, HPV, and influenza vaccines (19). The relatively lower molecular weight of the polysorbate 80 (1310 Da) in comparison to PEG (2000 Da) and the fact that polysorbate 80 is being used in many vaccines, monoclonal antibodies, and most of the injectable biological reagents for many decades alleviates its role as a possible trigger of anaphylaxis (18, 19).

SARS-CoV-2 vaccines are successful in reducing the severity of the disease, hospitalization, and mortality (20). The safety of SARS-CoV-2 vaccines has been successfully and carefully monitored prior to their authorization. However, the tracking of side effects that were not detected during clinical trials is still needed. The current study aimed to screen the incidence of local, systemic, and allergic reactions following SARS-CoV-2 vaccination. We also intended to screen possible factors that may affect the incidence and severity of post-vaccinal reactions, including previous COVID-19 infection, age, sex, and other immunocompromising conditions.

## Methods

### Participants

Vaccination was an inclusion criterion for filling the questionnaire. The questions seek the following information: whether participants are suffering from any chronic diseases or immunosuppressive disorders, whether they suffered from a previous SARS-CoV-2 infection or not, and the type and number of doses of the vaccine. The questionnaire included a leading question asked about the presence of absence of the side effects following vaccination. In case they have symptoms, they were asked about the individual side effects they experienced after the first and the second doses of the vaccines in separate sets of questions. There were three main clusters of questions including those related to local, allergic, and systemic reactions in addition to other unlisted symptoms. A question about the intensity of reaction or the severity of side effects was included; it graded the severity into mild (minor reactions that extended for ≤1–2 days), moderate (tolerable reactions extended to 2–3 days), severe (exacerbation of the post-vaccinal reactions in barely tolerable manner extended for ≥3 days but did not require hospitalization), and critical (adverse signs were severe enough to require hospitalization). We also asked about the duration of the post-vaccination side effects till the 7th day post vaccination and provided an option if the signs continued for more than 7 days. Also included was an open question so the respondent could write any other sign not listed in the questions. A link to the online questionnaire was distributed in different social media including Twitter, Snapchat, and WhatsApp.

### Statistical Analysis

The differences between the two vaccinated groups (BNT162b2 vs. those who received ChAdOx1) were compared using Chi square and Mann Whitney tests. The differences between the severity of the post-vaccinal reaction after the first and second BNT162b2 vaccination doses were also compared. The percentage of vaccinees experiencing side effects after having received the vaccine were calculated. All vaccinees were included in the adverse effects' analysis, including those who received a single dose of BNT162b2 or ChAdOx1. The probability of having severe side effects following the first and second BNT162b2 doses were also compared. Roc curve and logistic regressions were used for each of the specified variables to investigate whether adverse effects varied among different groups, and whether the previous exposure to COVID-19 infection could alter the response. Area under the curve (AUC) ≤ 0.5 poor discrimination or not useful, 0.7–0.8 is considered a good or an acceptable value, while 0.8–0.9 is very good value, while ≥0.9 is an excellent (21, 22).

## Results

### Demographic Characteristic of the Studied Subjects

The total number of users who reported vaccination with the BNT162b2 vaccine (2,601:1,846 females and 755 males) were higher than those who reported getting vaccinated with ChAdOx1 (1,569:1,028 females and 541 males). This included slightly younger individuals and they were more frequently female than users who reported receiving the ChAdOx1 inoculation (Table 1). A majority of respondents were 20–30 years old (44.29% (1152/2601), 51.8% (813/1569) for BNT162b2 and ChAdOx1, respectively). The study found a small number of respondents above 50 years old; 7.46% (194/2601) and 5.54% (87/1569), for BNT162b2 and ChAdOx1, respectively (Table 1).

**Table 1:** Frequencies and characteristics of the subjects in the current study.

**Variable**	**BNT162b2 n:2601 (55.22)**	**ChAdOx1 n:1569 (37.63)**	**Total n: 4170**	***P*-value**
**Sex, n (%)**				
Female	1,846 (70.97)	1,028 (65.52)	2,874(68.92)	0.001
Male	755 (29.03)	541 (34.48)	1,296(31.08)	
**Age groups, n (%)**				0.001
<20	476 (18.3)	195(12.42)	671(16.09)	
20–30	1,152 (44.29)	813 (51.82)	1,965 (47.12)	
31–40	410 (15.76)	261 (16.63)	671 (16.09)	
41–50	369 (14.19)	213 (13.57)	582 (13.96)	
51–60	156 (5.99)	71 (4.53)	227 (5.44)	
> 60	38 (1.46)	16 (1.02)	54 (1.29)	
**Nationality, n (%)**				
Saudi	2,484 (95.50)	1,416 (90.25)	3,900 (93.53)	0.001
Not Saudi	117 (4.50)	153 (9.75)	270 (6.47)	
**BMI, n (%)**				
<18.5	287 (11.03)	149 (9.50)	436 (10.46)	0.041
18.5–24.9	1,066 (40.98)	655 (41.75)	1,721 (41.27)	
25–29.9	682 (26.22)	420 (26.77)	1,102 (26.43)	
30–34.9	356 (13.68)	217 (13.83)	573 (13.74)	
35–39.9	156 (5.99)	75 (4.78)	231 (5.54)	
≥40	54 (2.08)	53 (3.37)	107 (2.57)	
**Previous infection with COVID-19, n (%)**				
No	2,283 (77.77)	1,390 (88.59)	3,673 (88.08)	0.868
Yes	318 (12.23)	179 (11.41)	497 (11.92)	
**The presence of chronic diseases, n (%)**				
No	2,152 (82.73)	1,295 (82.54)	3,447 (82.66)	0.43
Yes	449 (17.26)	274 (17.47)	723 (17.34)	
DM	164 (36.53)	126 (45.98)	290 (40.11)	
Hypertension	176 (39.20)	110 (40.15)	286 (39.56)	
DM and HTN	27 (6.01)	2 (0.73)	29 (4.01)	
Asthma	24 (5.35)	13 (4.74)	37 (5.12)	
Cardiac diseases	27 (6.01)	7 (2.55)	34 (4.7)	
Liver diseases	8 (1.78)	2 (0.73)	10 (1.38)	
Kidney diseases	8 (1.78)	6 (2.19)	14 (1.94)	
Cancer	8 (1.78)	1 (0.36)	9 (1.24)	
Multiple sclerosis	3 (0.67)	5 (1.82)	8 (1.11)	
Rheumatoid fever	4 (0.89)	2 (0.73)	6 (0.83)	

*Data are showed as number (percentage). Chi-square test was used to calculate the p-value for the difference between the first dose of BNT162b2 and ChAdOx1 vaccines*.

### Correlation Between the Severity of Post-vaccinal Reaction and Different Parameters

**Figure 1:**
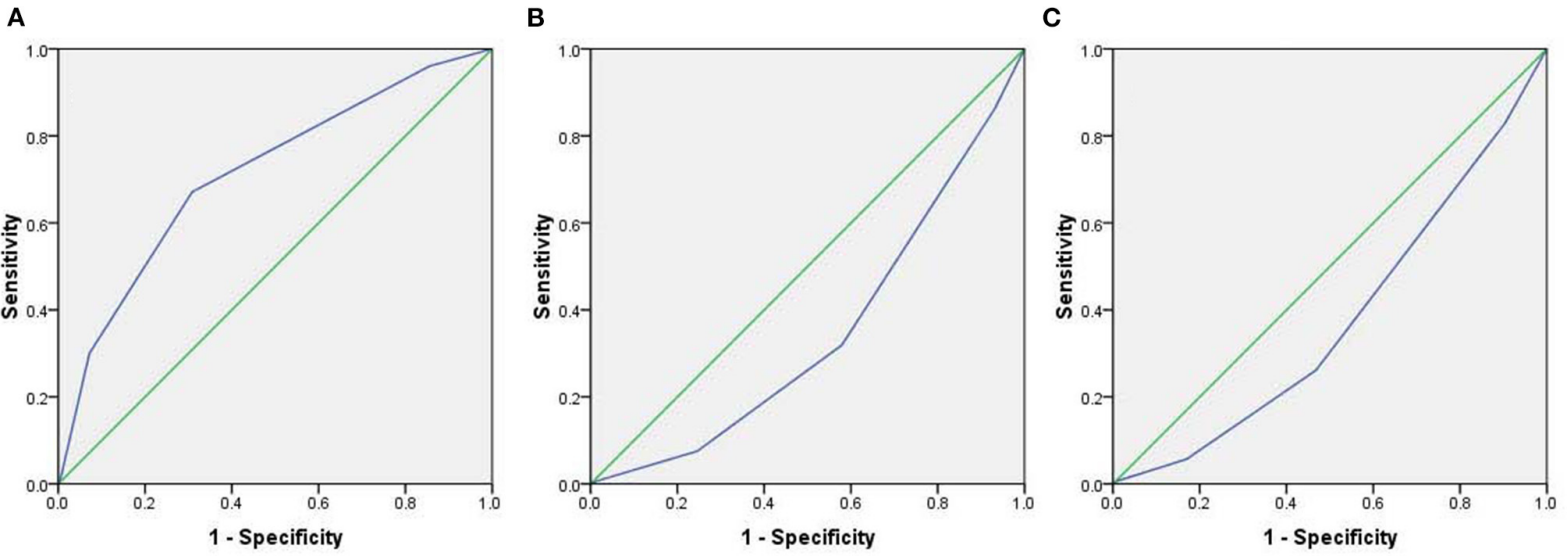
The Roc curve demonstrates the level of severity of post-vaccinal side effects and type of the vaccines. **(A)** ChAdOx1 vaccine, **(B)** A single dose of BNT162b2 vaccine, **(C)** Two doses of BNT162b2 vaccine.

We studied the correlation between the severity of the post-vaccinal reactions and age, sex, nationality, body mass index, previous COVID-19 infection, and the presence of chronic diseases in the vaccinated subjected. The presence of chronic diseases was found to show significant correlation to the development of post-vaccinal adverse effects (*P* < 0.016, *R* = 0.2). The female gender was also associated with the development of moderate and severe post-vaccinal adverse effects (*P* < 0.015, *R* = −0.133) (Table 2). There is a significant correlation between the type of vaccine and the severity of the post-vaccinal reaction *P* > 0.01, *R* = 0.381. The severe post vaccination side effects were found to be correlated with ChAdOx1 vaccine (Table 2). The Roc curve of the ChAdOx1 vaccine showed an acceptable good AUC value, 0.713, with the level of disease severity, 0.008 standard error and *P* < 0.001. While a single dose or two doses of BNT162b2 showed poor AUC of 0.347 and 0.378, respectively (Figure 1).

**Table 2:** Correlation of the severity of the post-vaccinal reactions and different parameters.

**Variable**	**Don't have side effect**	**Mild**	**Moderate**	**Severe**	**Critical**	**Significance and correlation**
**Sex, n (%)**						
Female	227 (7.9)	1,262 (43.9)	887 (30.9)	471 (16.4)	27 (0.9)	*P* < 0.015
Male	210 (16.2)	615 (47.5)	311 (24.0)	148 (11.4)	12 (0.9)	*R =* −0.133
**Age groups, n (%)**						
<20	82 (12.2)	277 (41.3)	207 (30.8)	96 (14.3)	9 (1.3)	*P* < 0.016
20–30	158 (8.0)	874 (44.5)	604 (30.7)	314 (16.0)	15 (0.8)	*R =* −0.069
31–40	69 (10.3)	316 (47.1)	177 (26.4)	105 (15.6)	4 (0.6)	
41–50	69 (11.9)	283 (48.6)	143 (24.6)	79 (13.6)	8 (1.4)	
51–60	41 (18.1)	105 (46.3)	58 (25.6)	21 (9.3)	2 (0.9)	
> 60	18 (33.3)	22 (40.7)	9 (16.7)	4 (7.4)	1 (1.9)	
**Nationality, n (%)**						
Saudi	410 (10.5)	1,776 (45.5)	1,113 (28.5)	565 (14.5)	36 (0.9)	*P* < 0.016
Non Saudi	27 (10.0)	101 (37.4)	85 (31.5)	54 (20.0)	3 (1.1)	*R =* 0.042
**BMI, n (%)**						
<18.5	42 (9.6)	197 (45.2)	125 (28.7)	68 (15. 6)	4 (0.9)	*P* < 0.0.16
18.5–24.9	162 (9.4)	744 (43.2)	520 (30.2)	282 (16.4)	13 (0.8)	*R =* −0.040
25–29.9	133 (12.1)	500 (45.4)	317 (28.8)	140 (12.7)	12 (1.1)	
30–34.9	56 (9.8)	274 (47.8)	149 (26.0)	89 (15.5)	5 (0.9)	
35–39.9	29 (12.6)	121 (52.4)	55 (23.8)	24 (10.4)	2 (0.9)	
≥40	15 (14.0)	41 (38.3)	32 (29.9)	16 (15.0)	3 (2.8)	
**Type of the vaccine**						
BNT162b2 (n:2601)	375 (14.42)	1,423 (54.71)	616 (23.68)	171 (6.57)	16 (0.62)	*P* < 0.01
ChAdOx1 (*n* = 1,569)	62 (3.95)	454 (28.93)	582 (37.09)	448 (28.55)	23 (1.47)	*R =* 0.381
**Previous infection with COVID-19, n (%)**						
No	385 (10.5)	1,681 (45.8)	1,040 (28.3)	528 (14.4)	39 (1.1)	*P* < 0.016
Yes	52 (10.5)	196 (39.4)	158 (31.8)	91 (18.3)	0 (0.0)	*R =* 0.035
**The presence of chronic diseases, n (%)**						
No	356 (10.3)	1564 (45.4)	1014 (29.4)	486 (14.1)	27 (0.8)	*P* < 0.016
Yes	81 (11.2)	313 (43.3)	184 (25.4)	133 (18.4)	12 (1.7)	*R =* 0.20

### Post-vaccinal Adverse Effects Following Administration of BNT162b2 or ChAdOx1

The severity of post-vaccinal side effects was found to be significantly higher in females in comparison to males (Table 3). ChAdOx1 vaccinees reported mild, moderate, severe, and critical side effects in 454/1569 (30.13%), 582/1569 (28.62%), 448/1569 (29.73%), and 23/1569 (1.53%), respectively. In contrast, mild side effects were recorded among the majority of BNT162b2 vaccinees 1423/2601 (63.92%) while moderate, severe, and critical side effects were 616/2601 (27.67%), 171/2601 (7.68%) and 16/2601 (0.72%), respectively (Table 4).

**Table 3:** The post vaccine adverse effects after vaccination in male and female subjects.

	**Female**		**Male**		
	**Number (2,874)**	**%**	**Number (1,296)**	**%**	***P*-value**
**Severity of the side effects**					
No side effects	227	7.9	210	16.2	0.001
Mild	1,262	43.9	615	47.5	
Moderate	887	30.9	311	24.0	
Severe	471	16.4	148	11.4	
Critical	27	0.9	12	0.9	
**Duration of the side effects**					
didn't have	250	8.7	216	16.7	0.001
1 day	699	24.3	425	32.8	
2 days	1,022	35.6	415	32.0	
3 days	492	17.1	132	10.2	
4 days	143	5.0	33	2.5	
5 days	84	2.9	22	1.7	
6 days	15	0.5	5	0.4	
7 days	57	2.0	10	0.8	
> 7 days	112	3.9	38	2.9	
**Local reactions, n (%)**					
Pain	2,490	86.6	945	72.9	0.001
Swelling	825	28.7	245	18.9	0.001
Swollen lymph nodes	112	3.9	30	2.3	0.009
**Systemic reactions, n (%)**					
Headache	1,353	47.1	456	35.2	0.001
Fever	1,256	43.7	524	40.4	0.048
Chill	759	26.4	279	21.5	0.001
Palpitation	377	13.1	85	6.6	0.001
Joint pain	1,161	40.4	447	34.5	0.001
Muscle pain	1,590	55.3	598	46.1	0.001
Fatigue	1,971	68.6	741	57.2	0.001
Impaired concentration	605	21.1	225	17.4	0.006
Insomnia	602	20.9	230	17.7	0.017
Dizziness	904	31.5	272	21.0	0.001
Diarrhea	238	8.3	77	5.9	0.008
Abdominal pain	259	9.0	72	5.6	0.001
Chest pain	333	11.6	80	6.2	0.001
Breathlessness	234	8.1	68	5.2	0.001
**Allergic reaction, n (%)**					
Rash	88	3.1	28	2.2	0.101
Itching	296	10.3	71	5.5	0.001
Swollen lips	64	2.2	16	1.2	0.031

*Data are showed as number (percentage). Chi-square test was used to calculate the p-value for the difference between the male and female subjects after the first dose of either BNT162b2 or ChAdOx1*.

**Table 4:** Comparison between the post vaccine adverse effects after vaccination with BNT162b2 and ChAdOx1 as well as those after the first and the second doses of BNT162b2.

	**First dose**			First and second doses of BNT162b2^*^		
	**BNT162b2**	**ChAdOx1**	***P*-value**	**1st dose**	**2nd dose**	***P*-value**
Number	2,601 (62.37)	1569 (37.63)	0.001	456 (100)	456 (100)	0.001
**Severity of the side effects**						
No side effects	375 (14.42)	62 (3.95)	0.001	78 (17.11)	63 (13.81)	0.149
Presence of side effects	2,226 (85.58)	1,507 (96.05)		378 (82.89)	392 (85.96)	
Mild	1,423 (63.92)	454 (30.13)	0.001	258 (56.57)	164 (35.96)	0.001
Moderate	616 (27.67)	582 (38.62)		94 (20.6)	135 (29.61)	
Severe	171 (7.68)	448 (29.73)		23 (5.04)	86 (18.85)	
Critical	16 (0.72)	23 (1.53)		3 (0.66)	6 (1.32)	
**Duration of the side effects**						
didn't have	375 (14.42)	62 (3.95)	0.001	78 (17.11)	63 (13.81)	0.246
1 day	679 (30.50)	474 (31.45)		132 (34.92)	126 (32.14)	
2 days	877 (39.40)	560 (37.16)		156 (41.27)	157 (40.05)	
3 days	366 (16.44)	258 (17.12)		59 (15.61)	61 (15.56)	
4 days	100 (4.49)	76 (5.04)		10 (2.64)	18 (4.59)	
5 days	52 (2.34)	54 (3.58)		7 (1.85)	4 (1.02)	
6 days	11 (0.49)	9 (0.60)		1 (0.26)	3 (0.77)	
7 days	44 (1.98)	23 (1.52)		4 (1.06)	8 (2.04)	
> 7 days	97 (4.36)	53 (3.52)		9 (2.38)	15 (3.83)	
**Local reactions, n (%)**						
Pain	2,088 (80.27)	1,347 (85.85)	0.001	355 (77.85)	334 (73.57)	0.132
Swelling	677 (26.03)	393 (25.05)	0.482	132 (28.95)	137 (30.18)	0.658
Swollen lymph nodes	98 (3.77)	44 (2.80)	0.097	22 (4.82)	34 (7.49)	0.094
**Systemic reactions, n (%)**						
Headache	861 (33.10)	948 (60.42)	0.001	143 (31.36)	194 (42.73)	0.001
Fever	617 (23.72)	1163 (51.75)	0.001	103 (22.59)	236 (51.98)	0.031
Chill	303 (11.65)	735 (46.85)	0.001	46 (10.09)	131 (28.85)	0.001
Palpitation	193 (7.42)	269 (17.14)	0.001	19 (4.17)	60 (13.22)	0.001
Joint pain	735 (28.26)	873 (55.64)	0.001	28 (6.17)	112 (24.56)	0.001
Muscle pain	1,088 (41.83)	1,100 (70.11)	0.001	31 (6.83)	162 (70.11)	0.001
Fatigue	1,358 (52.21)	1,354 (86.30)	0.001	217 (47.59)	198 (43.61)	0.229
Impaired concentration	367 (14.11)	463 (29.51)	0.001	46 (10.09)	103 (22.69)	0.001
Insomnia	402 (15.46)	430 (27.40)	0.001	54 (11.84)	43 (9.47)	0.247
Dizziness	526 (20.2%)	650 (41.4%)	0.001	62 (13.6%)	249 (54.8%)	0.001
Diarrhea	164 (6.31)	151 (9.62)	0.001	19 (4.17)	308 (67.84)	0.001
Abdominal pain	182 (6.99)	149 (9.50)	0.004	14 (3.07)	82 (18.06)	0.001
Chest pain	204 (7.84)	209 (13.32)	0.001	20 (4.39)	33 (7.27)	0.063
Breathlessness	149 (5.73)	53 (9.75)	0.001	8 (1.75)	18 (3.96)	0.045
**Allergic reaction, n (%)**						
Rash	75 (2.88)	41 (2.61)	0.067	9 (1.97)	12 (2.64)	0.501
Itching	233 (8.96)	134 (8.54)	0.645	33 (7.24)	42 (9.25)	0.269
Swollen lips	48 (1.85)	32 (2.04)	0.658	10 (2.19)	8 (1.76)	0.654

*Data are showed as number (percentage). Chi-square test was used to calculate the p-value for the difference between the first dose of BNT162b2 and ChAdOx1 or comparing the first dose of BNT162b2 with the second dose*.

* *The people who got the second dose of the vaccine (n = 456) were extracted from the total number of Pfizer-BioNTech vaccinees (n = 2601)*.

Most respondents to this survey about vaccine side effects suffered from one or more side effects while only 14.42% (375/2601) and 3.95% (62/1569) did not record any side effect for BNT162b2 and ChAdOx1, respectively. The most common side effect was localized pain after vaccine injection: 2,088 (80.27%), 1,347 (85.85%) for BNT162b2 and ChAdOx1, respectively. For ChAdOx1, fatigue 1,354 (86.30%), muscle pain 1,100 (70.11%), and headache were the common complaints. Meanwhile, for BNT162b2, fatigue 1,358 (52.21%), muscle pain 1,088 (41.83%), and headache were also the common systemic complaints but were significantly lower than that detected for the ChAdOx1 vaccine. Insomnia 430 (27.40%), palpitation 269 (17.14%), chest pain 209 (13.32%), breathlessness 153 (9.75%), diarrhea 151 (9.62), and abdominal pain 149 (9.50) were recorded in respondents vaccinated with ChAdOx1, which was significantly higher than those reported for BNT162b2 (Table 4). The detected palpitation was not correlated to any of the reported chronic diseases related to cardiac diseases (Table 5). Fever, chills, palpitation, joint pain, and impaired concentration showed a highly significant variation among subjects vaccinated with ChAdOx1 (~2-fold or more of the percentage detected following BNT162b2 vaccination; Table 4). Other side effects, including allergies (itching, rashes, and swollen lips) were reported in both BNT162b2 and ChAdOx1 vaccinated subjects but there were no significant differences among the two groups. There were no significant differences between the vaccinees with regard to the allergic or local side effects. ChAdOx1 showed a higher percentage of local pain at the site of injection than BNT162b2. In contrast, subjects vaccinated with ChAdOx1 showed a highly significant increase in the duration of systemic reactions than those vaccinated with BNT162b2. About 11.96% (304/2601) and 13.7% (215/1569) showed side effects extended from 4->7 days, while 72.9% (1897/2601) and 82.1% (1288/1569) showed side effects extended from 1–3 days for BNT162b2 and ChAdOx1, respectively (Table 4).

**Table 5:** Relationship between palpitation and the presence of hypertension, and cardiac disease in the tested subjects.

		**Palpitation**		**Significance and correlation**
		**No**	**Yes**	
HTN	No	3,443	441	*P* < 0.97 *R =* 0.015
	Yes	265	21	
Cardiac disease	No	3,678	458	*P* < 0.636 *R =* 0.007
	Yes	30	4	
HTN and DM	No	4,220	461	*P* < 0.216 *R =* 0.022
	Yes	28	1	

### Post-vaccinal Adverse Effects Following Administration of First and Second Doses of BNT162b2

Interestingly, subjects who received the second dose of BNT162b2 showed more moderate and severe reactions in a highly significant manner in comparison to the same subjects after the first dose of the vaccine (*P* < 0.001). No significant variation in local pain or post-vaccinal allergic reactions or even the duration of side effects was detected among respondents got the first and second dose of BNT162b2. Significant systemic reaction variations were more pronounced among subjects after getting the second dose in comparison to the same subjects after getting the first dose of the vaccine (Table 4). In contrast, the complaint of joint and muscle pain was more frequently detected after the first dose, as compared to the second dose. Diarrhea (308/456, 67.84%) and muscle pain (162/456, 70.11%) were very prominent complaints in a majority of the subjects who received the second dose in comparison to those got the first dose (19/456, 4.17%) (31/456, 6.83%), respectively (Table 4).

### Uncommon Post-vaccinal Adverse Effects Added by the Participants

The studied subjects reported uncommon post-vaccinal adverse effects, with a relatively considerable number of abnormal menstrual cycle, including increase in the time or increase in the pain or the bleeding, which was higher in BNT162b2 (18 cases) than in ChAdOx1 (7 cases). Other recorded signs included sore throat and/or dry mouth in 12 cases vaccinated with BNT162b2 and 3 cases vaccinated with ChAdOx1. Other rare signs were reported: anxiety, depression, sleepiness, mood disturbance, numbness of face, bruises in the leg, deep vein thrombosis, hypertension, pain in the testes, pain in the eyes, blurred vision, pain in the ear, and tinnitus (Table 6).

**Table 6:** Uncommon post-vaccinal adverse effects reported by the participants.

	**BNT162b2**		**ChAdOx1**
	**1st dose N (%)**	**2nd dose N (%)**	**1st dose N (%)**
Abnormal menstrual cycle	18 (0.69)	1 (0.22)	7 (0.45)
Anxiety	1 (0.04)	0 (0.00)	1 (0.06)
Depression	2 (0.08)	0 (0.00)	1 (0.06)
Mood disturbance	2 (0.08)	0 (0.00)	1 (0.06)
Numbness of face	1 (0.04)	0 (0.00)	0 (0.00)
Sore throat/dry mouth	12 (0.46)	3 (0.66)	3 (0.19)
Bruises in the leg	2 (0.08)	0 (0.00)	1 (0.06)
Deep venous thrombosis	1 (0.04)	0 (0.00)	0 (0.00)
Hypertension	2 (0.08)	3 (0.66)	0 (0.00)
Pain in testis	0 (0.00)	1 (0.22)	1 (0.06)
Pain in eyes	4 (0.15)	0 (0.00)	2 (0.13)
Blurred vision	1 (0.04)	0 (0.00)	1 (0.00)
Pain in ear	1 (0.04)	1 (0.22)	1 (0.06)
Tinnitus	0 (0.00)	0 (0.00)	2 (0.13)

## Discussion

In this study, the majority of respondents were 20–30 years old for both BNT162b2 and ChAdOx1. Considerable adverse side effects among respondents under 50 years of age, were reported. Previous studies support our findings as they showed that a higher rate of post-vaccinal side effects occurred among young subjects (23–25).

Subjects who suffered from chronic diseases are assumed to possess a weakened responsiveness to immunogens and hence are assumed to experience reduced side effects following vaccines (26, 27). In this study, chronic diseases were found to show significant correlation to the development of post-vaccinal adverse effects. However, no published information is available regarding these clusters of patients and their responsiveness or side effects following different COVID-19 vaccinations.

Subjects who previously experienced COVID-19 infection were reported to develop more severe side effects after vaccination (28). However, in our study, we did not find a considerable correlation between previous SARS-CoV-2 infection and the severity of post-vaccinal side effects.

In this study, the female gender was also associated with development of moderate and severe post-vaccinal adverse effects, showing a positive correlation of gender to the severity of the post-vaccinal side effects. This finding also agrees with other findings and confirms that the development of post-vaccinal side effects were significantly higher in females than in males (23, 24, 29). However, it disagrees with a recent study (30) that reported that males developed more severe post-vaccinal reactions in comparison to females.

In general, ChAdOx1 showed a highly significant increase in the severity and duration of systemic post-vaccinal reactions than BNT162b2. Meanwhile, the side effects of BNT162b2 after the second dose were more severe than that reported after the first dose of the vaccine. In this respect, our findings agree with other studies that reported similar findings (24, 31, 32).

The most common side effect was local pain after vaccine injection: 80.27% and 85.85% for BNT162b2 and ChAdOx1, respectively. This finding agrees with the data of phase 2/3 clincal trial that reported 88% pain at the site of injection following vaccination with ChAdOx1 (25), in individuals 18–55 of age and it reached >80% in individuals (≥16 years old) vaccinated with BNT162b2 (23). Similarly, the detected fatigue, muscle and joint pain following BNT162b2 and ChAdOx1 vaccinations matched what was reported previously (23, 25).

In the USA, many neurological side effects were reported (33, 34). In the current study, signs of transient central nervous system affections ranged from 14.1 to 29.5% of the reported side effects. They included impaired concentration, insomnia, and dizziness. These affections were reported in a significantly higher frequency in ChAdOx1 as compared to BNT162b2. Similarly, their frequencies were higher after the second dose of BNT162b2, in comparison to those after the first dose. Meanwhile, rare solitary neurological signs, including anxiety, depression, sleepiness, mood disturbance, numbness of face, pain in the ear, and tinnitus were also detected in the current study. Our finding agrees with previous reports of rare reports of tremor, diplopia, dysphonia, seizures, tinnitus, herpes reactivation, facial palsy, transverse myelitis, and acute disseminated encephalomyelitis detected in the USA following COVID-19 vaccination (33, 34).

Palpitation was not previously reported among the side effects of COVID-19 vaccination; however, a case report of postural orthostatic tachycardia syndrome was recently reported following BNT162b2 vaccination (35). Interestingly, in the current study, palpitation was reported with high frequency after COVID-19 vaccination: 7.42% and 17.14% for BNT162b2 and ChAdOx1, respectively. Similarly, the signs were reported more frequently in vaccinees after the second dose (13.22%) in comparison to the first dose (4.17%) for BNT162b2. In addition, chest pain and breathlessness were also reported in this study with ChAdOx1 and to a lower frequency among BNT162b2 vaccinees. In April 2021, the Vaccine Adverse Event Reporting System (VAERS) detected more than 1,000 cases reporting myocarditis and pericarditis following mRNA vaccination (36). Palpitation was also reported in another study in Saudi Arabia in 7(0.4%) subjects following vaccination with ChAdOx1 (30) and in South Korea in 28.3% and 4.3% of ChAdOx1 and NT162b2 vaccinees, respectively (37).

Diarrhea and abdominal pain were recorded in respondents vaccinated with ChAdOx1. The percentages of such side effects were significantly higher than those reported for BNT162b2. The latter showed a higher frequency of complaints after the second dose in comparison to the first dose. A meta-analysis study reported similar side effects for both BNT162b2 and ChAdOx1 (38).

Menstrual disturbance, including excessive hemorrhage and irregular menstrual cycle were reported after the administration of BNT162b2 (643 cases) and ChAdOx1 (315 cases) (39). In contrast, in this study, irregular menstrual cycle was reported in 18 cases vaccinated with BNT162b2 (0.69%) and only 7 cases (0.45%) vaccinated with ChAdOx1.Thrombocytopenia and underlying platelets disorders, or possible hormonal disturbance may explain excessive menstrual bleeding. More investigations are needed to determine the link between COVID-19 vaccination and the menstrual irregularities (40, 41).

## Conclusion

In conclusion, both vaccines induce transient side effects that ranged from mild to severe. ChAdOx1 induced more severe side effects than BNT162b2. The latter vaccine induces more severe adverse effects after the second dose of the vaccine. High frequency of palpitation was reported following vaccination. All the reported side effects are tolerable. Women are more prone to developing more severe side effects and for longer durations. Palpitation is among the common systemic side effects that appear following vaccination of both vaccines.

### Limitation of the Study

The results of this questionnaire are self-reported from those receiving the vaccine and not clinically confirmed by physicians as it may impact result reporting due to differences in interpretation and tolerance thresholds from patient to patient. The study is limited by possible information bias, including misclassification and the duration description of some important manifestations like palpitation and chest pain. The utilization of social media may bias the cohort toward age demographics most familiar with the technology (as evidenced by the high numbers of 20–30-year-olds and a lack of 50+ year old patients) and socioeconomic demographics with routine access to such platforms.

## Data Availability Statement

The original contributions presented in the study are included in the article/supplementary material, further inquiries can be directed to the corresponding author/s.

## Ethics Statement

The studies involving human participants were reviewed and approved by Taif University Ethical Committee with approval No. 42-169 on 3/05/2021.

## Author Contributions

ASA, ANA, MA, and DA collected, curated, analyzed the data, and wrote the first draft of the manuscript. AA-M conceptualized the study, verified the results, and critically revise the manuscript. All authors reviewed and edited revisions of the manuscript.

## Funding

This work was funded by Taif University Deanship of Scientific Research (Project No. 1-441-41).

## Conflict of Interest

The authors declare that the research was conducted in the absence of any commercial or financial relationships that could be construed as a potential conflict of interest.

## Publisher's Note

All claims expressed in this article are solely those of the authors and do not necessarily represent those of their affiliated organizations, or those of the publisher, the editors and the reviewers. Any product that may be evaluated in this article, or claim that may be made by its manufacturer, is not guaranteed or endorsed by the publisher.
